# Analysis of the Possibility of Plastic Deformation Characterisation in X2CrNi18-9 Steel Using Measurements of Electromagnetic Parameters

**DOI:** 10.3390/ma14112904

**Published:** 2021-05-28

**Authors:** Maciej Roskosz, Krzysztof Fryczowski, Lechosław Tuz, Jianbo Wu, Krzysztof Schabowicz, Dominik Logoń

**Affiliations:** 1 Faculty of Mechanical Engineering and Robotics, AGH University of Science and Technology, al. Mickiewicza 30, 30-059 Kraków, Poland; 2 Department of Power Engineering and Turbomachinery, Faculty of Energy and Environmental Engineering, Silesian University of Technology, Akademicka 2A, 44-100 Gliwice, Poland; kfryczowski@polsl.pl; 3 Faculty of Metals Engineering and Industrial Computer Science, AGH University of Science and Technology, al. Mickiewicza 30, 30-059 Kraków, Poland; ltuz@agh.edu.pl; 4 Department of Engineering Science and Mechanics, Sichuan University, Chengdu 610065, China; wujianbo@scu.edu.cn; 5 Faculty of Civil Engineering, Wrocław University of Science and Technology, Wybrzeże Wyspiańskiego 27, 50-370 Wrocław, Poland; krzysztof.schabowicz@pwr.edu.pl (K.S.); dominik.logon@pwr.edu.pl (D.L.)

**Keywords:** residual magnetic field, Barkhausen noise, LCR circuits, plastic deformation, austenitic steel

## Abstract

An analysis was conducted on the possibility of making an assessment of the degree of plastic deformation ε in X2CrNi18-9 steel by measuring three electromagnetic diagnostic signals: the Barkhausen noise features, the impedance components in in-series LCR circuits, and the residual magnetic field components. The impact of ε on a series of different extracted features of diagnostic signals was investigated. The occurrence of two regions of sensitivity was found for all the features of the analysed signals. The two regions were separated by the following critical deformation value: ε ~ 10% for the components of the residual magnetic field and ε ~ 15% for the normalised components of impedance. As for the Barkhausen noise signal, the values were as follows: ε ~ 20% for the mean value, ε ~ 20% for the peak value of the signal envelope, and ε ~ 5% for the total number of the signal events. Metallographic tests were performed, which revealed essential changes in the microstructure of the tested material for the established critical values. The martensite transformation occurring during the plastic deformation process of X2CrNi18-9 austenitic steel process generated a magnetic phase. This magnetic phase was strong enough to relate the strain state to the values of diagnostic signals. The changes in the material electromagnetic properties due to martensitic transformation (γ → α’) began much earlier than indicated by the metallographic testing results.

## 1. Introduction

Research is now being conducted in many scientific centres on methods that will enable the determination of the effect of cold plastic strain [1,2,3,4], mechanical fatigue [5,6,7], heat treatment [8], and creep [9] on the state and electromagnetic properties of austenitic steels. The applied diagnostic signals are the quantities describing the magnetic hysteresis loop, the eddy currents, the Barkhausen noise parameters, and the changes in the anisotropy of electromagnetic properties. The variations in these parameters result from the state of the microstructure, the grain size, and the impact of the dislocation density on the material electromagnetic properties.

Austenitic steels are widely used materials, and the strain-induced martensite transformation occurring in them, depending on the chemical composition, the magnitude of the rolling reduction, and the deformation temperature can have both favourable effects causing the material strengthening (a higher yield point or an increase in tensile strength) and unfavourable consequences causing a decrease in corrosion resistance and the appearance of the ferromagnetic phase [10,11,12,13,14,15]. Under the influence of cold plastic strain, a change occurs in the dislocation structure. As a result, metastable austenite undergoes a partial transformation into martensite ε and ferromagnetic martensite α’ with a body-centred cubic lattice [12,13].

Novotný et al. [1] introduced a novel application of magneto-optical films. At the magnetic field sensitivity of 100 A/m, coercivity can be mapped with a resolution as high as 50 μm. Promising results were obtained for austenitic steel by applying the magneto-optical method to indicate critically degraded (plastically deformed) locations.

O’Sullivan et al. [2] characterised work hardening of an austenitic stainless steel grade (SS404) using non-destructive magnetic measurement techniques, including measurements of the magnetic Barkhausen noise, the ferromagnetic phase, and coercivity. It was found that the material work-hardening was caused by the dislocation density rather than by the α′-martensite phase. The coercivity measurement proved to be a useful non-destructive quantitative method for characterizing work hardening in relation to the degree of plastic deformation.

In [3], the authors investigated selected phase transformations of the AISI 304 austenitic steel. The Barkhausen noise, coercivity, and ferrite content were measured to identify changes in the strain-induced α′-martensite phase due to cold rolling and elongation. The research proved that it was possible to study the mechanism of austenite transformation into the α′ phase, and the reverse transformation of the α′ phase into austenite.

In [4], the authors presented experimental studies on the amount of transformed martensite by measuring the continuous change in impedance during plastic deformation on specimens made of the 304 steel grade. The specimens were cores of a prototype solenoidal coil, which was subjected to compressive load.

In [5], the authors investigated specimens of the chromium-nickel steel used to make the generator retaining rings and the generator rotor shrouding. The specimens were subjected to fatigue and static loads. The austenite instability became apparent after plastic deformation (increase in the material permeability by about 0.1 μ_r_). Magnetic measurements based on austenite instability detection in mechanical and thermal correlations are an alternative to ultrasonic wave attenuation tests. Moreover, they give a more complete picture of the wear degree of the retaining ring (material degradation evaluation).

Vincent et al. [6] investigated the low-cycle fatigue (LCF) of steel 304L and the influence of the strain-induced α′-martensite on the magnetic Barkhausen noise (BN). It was shown that the variations of the martensite content induced by LCF could be related to and characterised by the BN. The number of cycles had an effect on the α′-martensite phase, and the α′ peak was clearly visible in the BN signal envelope.

In [7], AISI 31 austenitic stainless steel samples were subjected to fatigue testing. The effect of fatigue on the accumulation of damage and changes in the content of the α′-martensite was investigated. The obtained results showed the possibility of assessing the fatigue state of the AISI 31 steel using acoustic nonlinearity measurements and magnetic coercivity.

In [8], the authors investigated the relationship between the eddy current output signal and the surface hardness of a martensitic AISI 410 stainless steel sample in terms of impedance and inductance. They also examined the effects of different quenching temperatures on the steel surface hardness.

Augustyniak et al. [9] tested samples of 347, 321, and 304 austenitic steels taken from service-aged power plant boiler tubing. The accumulation of damage due to the creep process was proportional to the concentration of the created magnetic oxide layer. Simultaneously, a magnetic ferrite phase also formed in the grains and at grain boundaries under the scale layer. The content of the ferrite-phase layer was proportional to the initial creep-related damage. These changes were related to the eddy current signal.

This paper is focused on the analysis of the possibility of characterizing the plastic—strain ratio in specimens made of X2CrNi18-9 steel by measuring the residual magnetic field, the impedance components in in-series LCR circuits, and the Barkhausen noise. The same measurement quantities were used to characterise the active stress state in [16]. Additionally, metallographic tests were performed to observe the structural changes occurring due to deformation during static tensile testing.

## 2. Theoretical Basics

The authors of this paper have often used magnetic methods of non-destructive testing to solve various problems related to the broadly understood characterisation of the material state. For this reason, the theoretical foundations have already been presented many times. A synthetic description of the theoretical basis related to this article is included in [16], where an analysis was conducted of the possibility of assessing active stresses in steel elements by measuring electromagnetic diagnostic signals. Many articles were referred to in [17,18,19,20,21,22,23,24,25,26,27,28,29,30,31,32,33,34,35], where information can be found on the theoretical basis of the applied electromagnetic methods.

In chromium-nickel alloy steels, e.g., X5CrNi18-10, X2CrNi18-9, or X18CrNiSi18-9, depending on the concentration of Cr and Ni and other alloying elements (e.g., Mo, Mn, N, Si), an austenitic structure occurs in the supersaturated state at room temperature. The austenitic structure gets stabilised with an increase in Ni and other austenitic elements. In addition, the structure may also contain some ferromagnetic ferrite δ arising due to the conditions of steel crystallization. Depending on the amount of alloying elements, the ferrite content may increase up to several percent. To evaluate the structure and compactness of the magnetic phase, equivalents of austenite- and ferrite-forming elements can be used. This indicates that the chemical composition of steel itself has a direct impact on the content of the magnetic phase, which can be changed, usually increased, by heat treatment, strain due to cold or hot working, or by welding processes.

Austenitic Fe-Cr-Ni steels in the post-supersaturation state retain the austenitic structure (γ-phase) with a face-centred cubic (fcc) lattice. Depending on the chemical composition, austenite can be a metastable phase undergoing martensite transformation at cooling below temperature M_s,_ or due to critical plastic deformation at temperatures higher than M_s_. Due to the chemical composition, temperature M_s_ is lower than room temperature, which ensures high durability of austenite. Austenitic steels are commonly used materials, and the strain-induced martensite transformation occurring in them can have both favourable effects strengthening the material and unfavourable consequences resulting in a decrease in their corrosion resistance and the appearance of the ferromagnetic phase [10,11,12,13].

Due to cold plastic strain, austenitic chromium-nickel steels undergo significant strengthening depending on their chemical composition, the magnitude of the rolling reduction, and the deformation temperature. Under the influence of cold plastic strain, a change occurs in the dislocation structure. As a result, metastable austenite undergoes a partial transformation into martensite *ε* and ferromagnetic martensite α’ with a body-centred cubic lattice [12,13]. A similar transformation for austenitic steels occurs during quenching at low temperatures. It is believed that there are two possible mechanisms corresponding to such transformations [36,37]: one is the γ → ε → α’ transformation, where the ε phase is an intermediate phase with a closely packed hexagonal structure; in the other, a direct γ → α’ transformation is possible. Independently, the γ → ε transformation may also occur.

The factor deciding about the possibility of martensite ε formation is the austenite stacking fault energy (SFE), which depends on the steel chemical composition and the deformation temperature. Martensite ε can be created during cold plastic deformation if the SFE at room temperature is <30 MJm^−2^ [38]. The SFE parameter determines the type of the deformation (slip) system, which enables the formation of the intermediate ε phase or leads to the direct formation of the α’ phase. In steels with a higher SFE value, where the basic system of {111} austenite deformation occurs [39], the formation of the ε phase was not observed. However, it has been shown that, depending on the deformation conditions, in the same steel the transformation can occur directly or with the participation of the intermediate phase. The element, which strongly inhibits the γ → ε transformation, is nickel. In this case, the austenite-to-martensite transformation occurs directly γ → α’ [36], which means that, under small deformation below the critical deformation value, the changes occur only in the region of single grains, while above the critical point they will affect the whole cross-section.

## 3. Experimental Details

**Figure 1:**
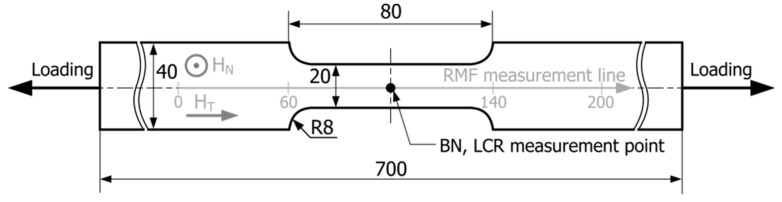
Tested specimen geometry with marked measuring points (RMF—residual magnetic field, H_N_—normal component of the RMF, H_T_—tangential component of the RMF, LCR—impedance components of the LCR measuring circuit, BN—Barkhausen noise).

**Figure 2:**
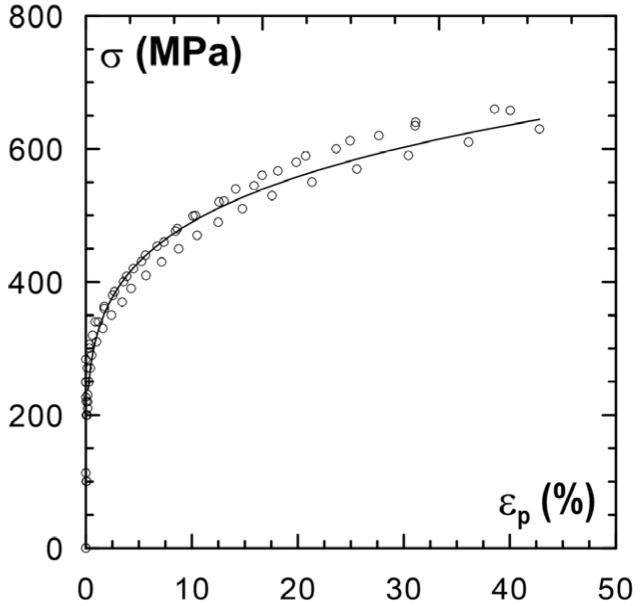
Relationship between engineering stress σ and plastic deformation ε_p_.

The testing was performed for flat specimens made of X2CrNi18-9 steel (chemical composition—cf. Table 1), whose initial geometry is shown in Figure 1. Figure 2 shows the relationship for the tested steel between the set value of engineering stress in the loading process and the plastic deformation value measured after the unloading of the specimens (between the 60th and the 140th measuring point). The specimens were subjected to static tensile loads to obtain appropriate plastic deformation.

The specimens were loaded using the Galdabini Sun 10P tensile strength testing machine (Galdabini, Cardano al Campo, Italy). The test conditions were as follows: temperature: 21 ± 2 °C and the traverse speed: 2 mm/min. After the set value of deformation was achieved, the specimens were unloaded and examined beyond the machine on the test stand, keeping the same orientation of the specimen in relation to the Earth’s magnetic field. Relative deformation was defined for an 80 mm long section of the specimen (between the 60th and 140th measuring point—cf. Figure 1). The initial distance between the points was 1 mm, but it increased with the rise in plastic deformation. The test was carried out for as-received samples with an elongation between 0% to 55% (no fracture). All the specimens, both in the as-received state and after plastic deformation, were tested using the MPD-100A magnetic field detector (R&J Measurement, Borowa, Poland). The results of the measurements of the magnetic phase are presented in Table 2. Of all the deformed specimens, fifty were selected for the testing of magnetic parameters, and the order of the measurements was as follows: residual magnetic field (RMF) components, LCR circuit impedance components, the Barkhausen noise. Metallographic testing was carried out only for selected specimens with 10%, 20%, and 40% elongation.

The TSC-1M-4 magnetometer (Energodiagnostika Co. Ltd., Moscow, Russia) was used for RMF measurements. For RMF, two components (H_N_—normal component, H_T_—tangential component measured in the direction parallel to the applied load) were measured using the TSC-2M (Energodiagnostika Co. Ltd., Moscow, Russia) measuring head. The measuring apparatus was calibrated in the magnetic field of the Earth, with the assumed value of 40 A/m. The measurements on the test stand were always carried out in the same place and with the same position of the specimen. The magnetic field components in the location where the measurements were performed had the following values: H_T_ = 8 A/m, H_N_ = 40 A/m. The magnetic field strength was measured along the measurement line (cf. Figure 1) in 200 points.

**Figure 3:**
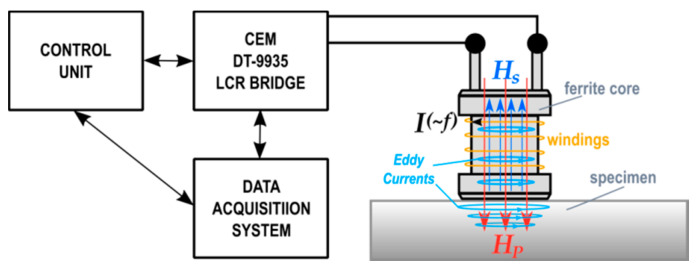
Measuring circuit diagram.

The system measuring the LCR components is schematically shown in Figure 3. It consists of a CEM DT-9935 automatic LCR bridge and a Fastron 09P-152J-50 choke coil (winding inductance 1.5 mH ± 5%, ferrite core with a diameter of 8.5 mm at the point of contact with the tested surface, resistance 1 Ω, test line resistance: 26.7 Ω).

Due to the magnetic coupling and the self-induction phenomenon, the characteristic values of the measuring coil change when it is applied to the specimen surface. The flow of electric current *I* with frequency *f* results in an alternating magnetic field *H_p_*. At the same time, eddy currents *I_EC_* are induced in the tested material, generating the *H_s_* field. The electromagnetic properties of the material affect the obtained values. The degree of plastic deformation causes a change in permittivity *ε*, relative magnetic permeability *μ_r_*, and conductivity *γ*. The changes in these quantities influence the resistance and the inductance value of the measuring coil coupled to the surface.

**Figure 4:**
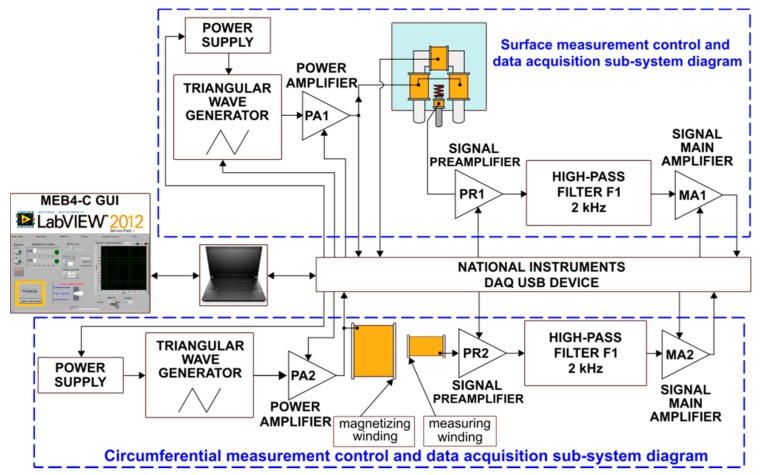
Schematic diagram of the MEB4-C system.

The Barkhausen noise was measured using the MEB4-C system (Mag-Lab, Gdańsk, Poland). The system diagram is shown in Figure 4. It enables measurements with the use of a surface measurement sub-system (upper part of Figure 4) and a circumferential measurement sub-system (lower part of Figure 4). The former was used during the testing. The measuring head contains a magnetic field excitation system and the Barkhausen noise signal detection system. The system configuration was as follows: sampling frequency—800 kHz, magnetizing current frequency—2.04 Hz, magnetizing current amplitude—200 mA, pre-amplifier gain PR1—× 1, main amplifier gain MA1—35 dB.

The Barkhausen noise was measured only in two directions: perpendicular (⊥) and parallel (∥) to the load. The choice of such directions of measurements results from the authors’ own research related to changes in hardness due to cold working [17] and from the literature on the influence of plastic deformation on the Barkhausen noise [40,41].

The absolute values of the Barkhausen noise parameters are calculated using the following Equation (1):

(1) $$V=\sqrt{V_{⊥}^{2}+V_{∥}^{2}}$$

where: $V_{⊥}$ and $V_{∥}$, respectively, are the parameter values for the two directions of the magnetizing field application.

The metallographic examination was conducted to verify the changes in the material indicated in the NDT test. To avoid the influence of the directionality of the ferrite bands, the tested material was sampled transversely to the rolling direction. The deformed part was sectioned using a precise cut-off machine (Struers, Willich, Germany), and intensive cooling was applied.

The metallographic observations were conducted using light microscopy (Leica LM/DM microscope—Leica, Wetzlar, Germany) and scanning electron microscopy (Phenom XL—Thermo Fisher Scientific, Waltham, MA, USA). The specimens were pre-ground using water abrasive paper and then polished and etched electrolytically (time: 10–15 s, current: 20 mA, voltage: 35 V) to avoid the influence of the effect of abrasive papers on the surface of the specimen. A cross-sectional microscopic examination was carried out after polishing and electrolytic etching in a 10% CrO_3_ water solution. Due to the applied preparation method, local etch defects, so-called etch pits, were observed on the surface of the metallographic specimens. XRD was performed in a D8 Advance Diffractometer (Bruker, Billerica, MA, USA) using Cu_Kα_ radiation; the magnetic phase (the content of the strain-induced martensite α’-phase) was measured using the MPD-100A magnetic field detector (R&J Measurement, Borowa, Poland).

Hardness measurements were performed using the Vickers method with an intender load of 10 kG (98.07 N), with a Zwick/Roell ZHU 187.5 hardness tester (Zwick Roell Group, Ulm, Germany).

**Figure 5:**
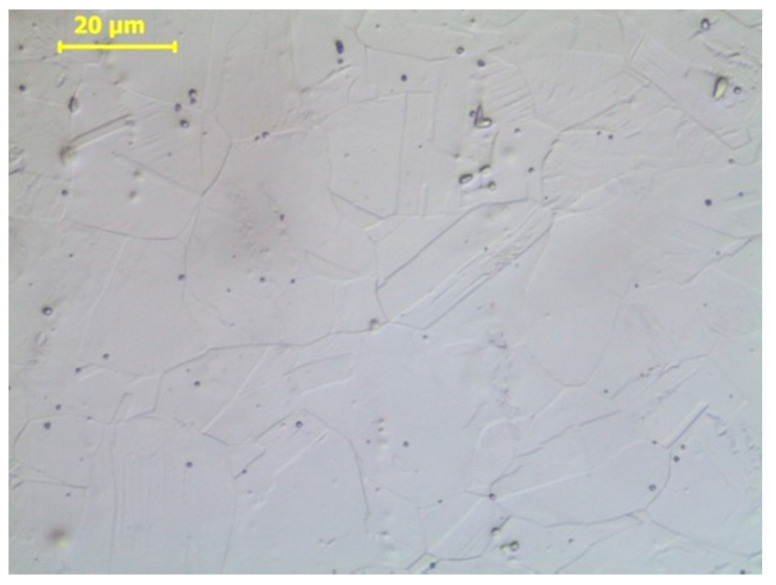
Austenitic structure of X2CrNi18-9 steel in the as-received state.

The tested steel was characterised by an austenitic structure, with a small content of ferrite δ arranged in the steel rolling direction. The structure showed only a few twin boundaries (annealing twins)—Figure 5. The testing of the steel magnetic phase content in the as-received state showed a value below 0.2%.

## 4. Analysis Results and Discussion

### 4.1. Residual Magnetic Field

**Figure 6:**
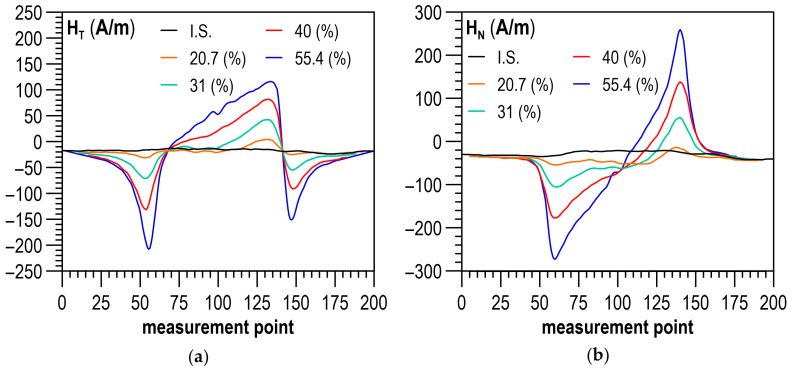
Distribution of the RMF components for different plastic deformation states: (**a**) tangential component H_T_; (**b**) normal component H_N_.

**Figure 7:**
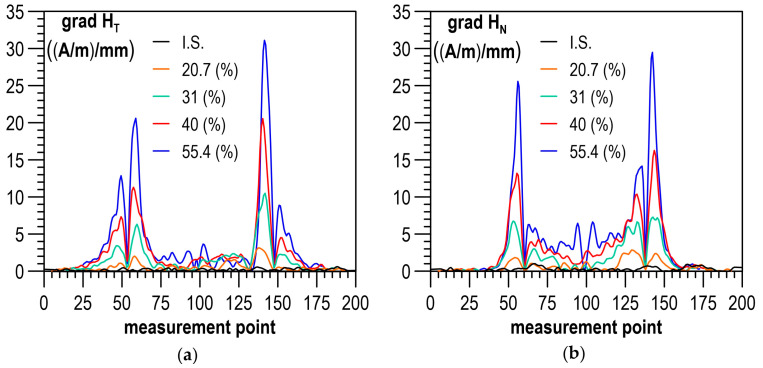
Distribution of the gradient of the RMF components for different plastic deformation states: (**a**) gradient of tangential component grad H_T_; (**b**) gradient of normal component grad H_N_.

Figure 6a,b shows the example distributions of the RMF components for the initial state (I.S.) and selected values of plastic strain. The plastically deformed region of the specimen with a smaller cross-section, lying between the 60th and the 140th measuring point, stands out in the distributions of both RMF components under analysis. As the plastic deformation degree got higher, the values of tangential component H_T_ increased, while the curves of normal component H_N_ in the plastically deformed region made an anticlockwise rotation. In the place of transition from the deformed to the non-deformed region, local extrema occurred of both tangential component H_T_ (Figure 6a) and normal component H_N_ (Figure 6b). They definitely took different values and had a different trend of changes compared to the rest of the sample. Due to the high variability of the values of the RMF components in the plastically deformed area, the further analysis of the measurement results aiming to develop a diagnostic relation was focused on the analysis of the gradients of the RMF components. The gradients of the changes in the RMF components (understood as absolute values of function derivatives) were determined by segmental approximation of the measurement results using third-degree spline functions. Example distributions of gradients, corresponding to the distributions of the RMF components, presented in Figure 6a,b, are shown in Figure 7a,b. The gradient distributions were dominated by two maxima in the zones of transition from the deformed to the non-deformed region.

An analysis was performed on the impact of the plastic deformation degree on the maximum values of gradients of the RMF components occurring in the transition zones and on the mean values of gradients of the RMF components determined for an area with a constant cross-section on the segment between the 90th and the 110th point on the specimen measurement line (cf. Figure 1).

**Figure 8:**
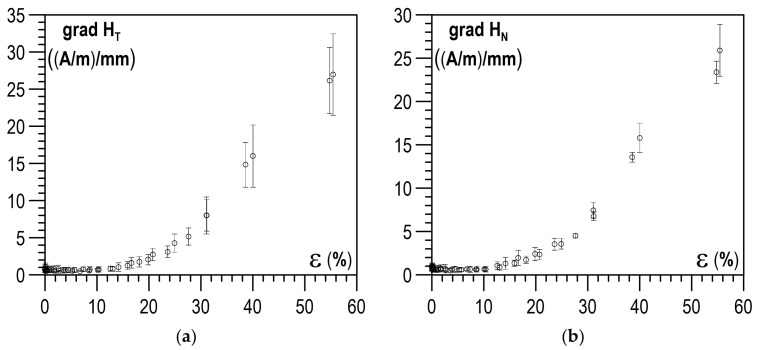
Relation between the maximum gradient value and the plastic deformation degree: (**a**) max gradient of tangential component grad H_T_; (**b**) max gradient of normal component grad H_N_.

**Figure 9:**
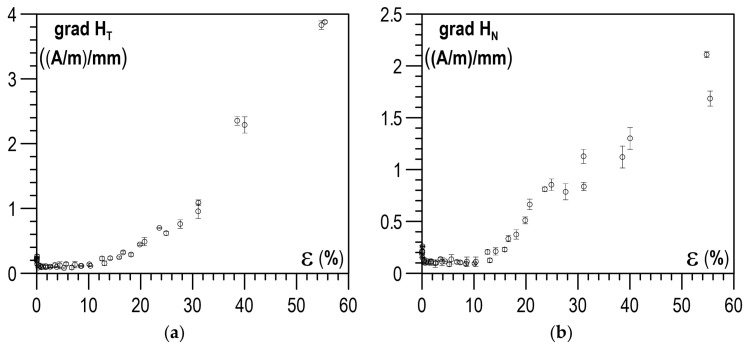
Relation between the mean gradient value and the plastic deformation degree: (**a**) mean gradient of tangential component grad H_T_; (**b**) mean gradient of normal component grad H_N_.

The relations determined for the X2CrNi18-9 steel specimens between the plastic deformation degree and the maximum gradients of the RMF components are shown in Figure 8a,b, whereas Figure 9a,b illustrates the relations between the plastic deformation degree and the mean gradients of the RMF components. As the deformation degree got higher, both maximum and mean values of the gradients of the RMF components increased. Unfortunately, for the tested X2CrNi18-9 steel, it could be seen that relatively unequivocal relations between the degree of plastic deformation and the values of the RMF gradients occurred only after the plastic deformation degree exceeded 10%. For lower plastic deformation values, the gradients of the RMF components did not change noticeably.

### 4.2. Components of Impedance of the In-Series LCR Circuit

**Figure 10:**
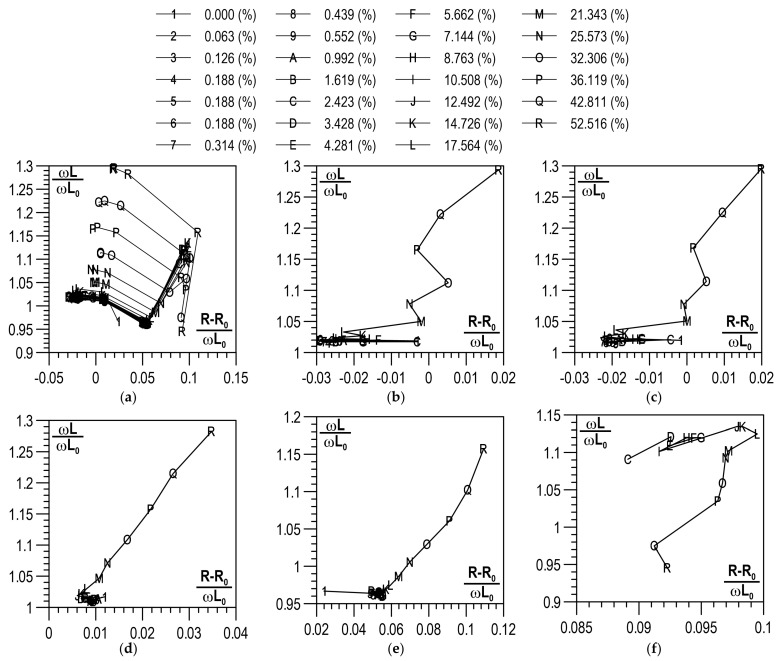
Results obtained from the analysis of the LCR measurements: (**a**) curves of normalised impedance components for different states of plastic deformation; distribution of normalised impedance components for: (**b**) f = 0.1 kHz; (**c**) f = 0.12 kHz; (**d**) f = 1 kHz; (**e**) f = 10 kHz; (**f**) f = 100 kHz.

For each plastic strain, value curves of normalised impedance components were developed. The curves in Figure 10a illustrate the normalised impedance components and, Figure 10b–f presents the distributions of normalised impedance components.

In Figure 10a–f, it can be seen that the effect of the plastic deformation degree on normalised components of impedance (R − R_0_)/ωL_0_, ωL/ωL_0_ becomes unequivocally visible only when plastic deformation reaches the level of about 15% and higher. For frequencies in the range from 0.1 kHz to 10 kHz, an increase in the plastic deformation degree was accompanied by a rise in the value of ωL/ωL_0_, while for the frequency of 100 kHz, ωL/ωL_0_ first increased until the plastic deformation degree reached the level of about 15% to show a decreasing trend later on. A constant trend in changes in the (R − R_0_)/ωL_0_ component occurred only at frequencies f equal to 1 kHz and 10 kHz for plastic deformation degrees higher than 15%.

### 4.3. Barkhausen Effect

**Figure 11:**
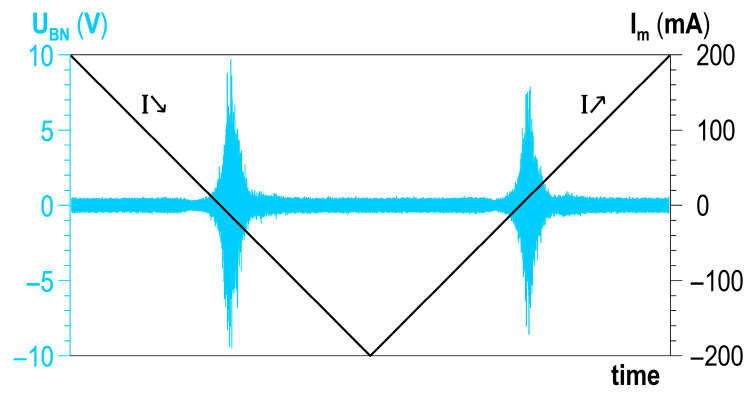
Detailed description of a single cycle of changes in magnetisation.

An analysis was performed of the possibilities of developing a correlation between the plastic deformation degree and the Barkhausen noise signal. The Barkhausen noise was measured for 10 magnetisation cycles. Two halves could be distinguished in a single magnetisation cycle (cf. Figure 11)—the first marked as I↘ (exciting current diminishes) and the second marked as I↗ (exciting current rises).

The following BN quantitative parameters were analysed: the RMS voltage value U_RMS_, the envelope of the BN signal (peak value), and the distribution of the total number of events NoE_TOT_. The formula for RMS voltage U_RMS_ was described in [16], and the distribution of the total number of events NoE_TOT_ was characterised in [16,17].

**Figure 12:**
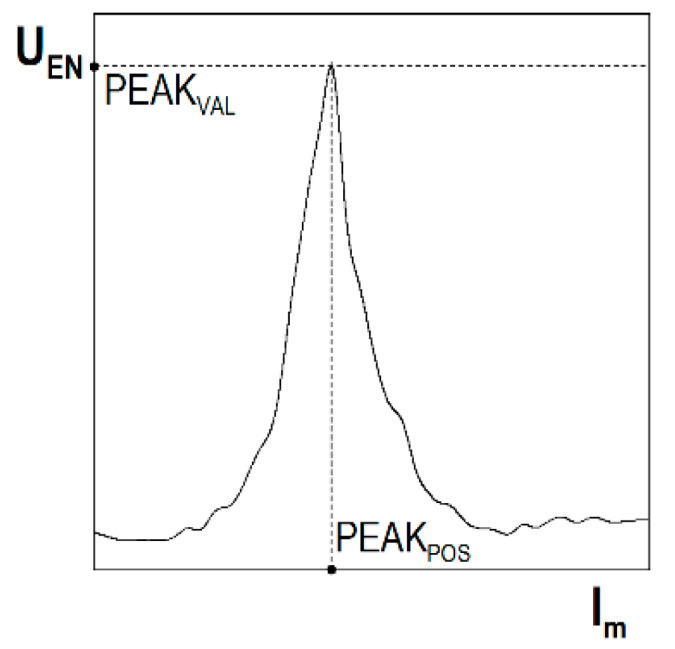
Characteristic quantities of the envelope.

The BN envelope was obtained through the smoothing averaging operation on the absolute values of the U_BNi_ voltage pulses. A multiple smoothing filtering operation using the Savitzky–Golay filter was used for this purpose. The envelope was characterised by local extrema, which could be described using their coordinates defining the magnetisation current PEAK_POS_ and the Barkhausen noise voltage PEAK_VAL_, which are graphically presented in Figure 12.

**Figure 13:**
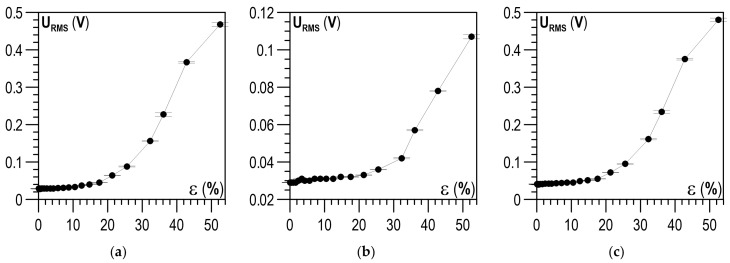
Relation between plastic deformation ε and U_RMS_: (**a**) parallel direction; (**b**) perpendicular direction; (**c**) module of directions.

The effect of the plastic deformation degree on the RMS value of voltage (U_RMS_) is shown in Figure 13a–c. Distinct changes could be observed in the U_RMS_ values from plastic deformation ε of about 20%, both for the parallel and the perpendicular direction (Figure 13a,b, respectively). However, for the latter, the U_RMS_ values were significantly lower compared to the parallel direction. The dependence of voltage U_RMS_ on ε for the module (Figure 13c) resembled both qualitatively and quantitatively the relation for the parallel direction (Figure 13a)—the values were substantially higher for the parallel direction and had a decisive impact on the value of the module.

**Figure 14:**
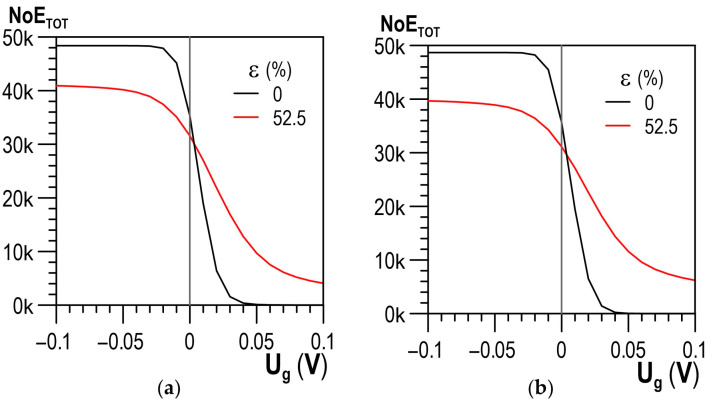
Distribution of the total number of events NoE_TOT_ depending on the threshold voltage for two strain states: (**a**) parallel direction; (**b**) perpendicular direction.

An analysis was conducted on the impact of plastic deformation ε on the values of the total number of events NoE_TOT_ for the entire range of discrimination voltage U_g_ between −10 V and 10 V. It was found that when approaching the value of 0 V on the side of negative voltage, a rise in plastic deformation caused a drop in the number of events. Close to 0 V on the side of positive voltage values, the opposite trend could be observed—as the degree of plastic deformation got higher, the number of events increased. This phenomenon occurred for both the parallel and the perpendicular direction of magnetisation (cf. Figure 14a,b, respectively). The influence of the magnetisation direction (the magnetic field direction in relation to the direction of the tensile force) was slight.

**Figure 15:**
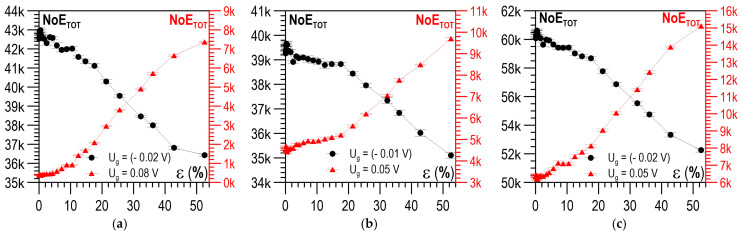
Relation between plastic deformation ε and mean NoE_TOT_ values for different values of threshold voltage U_g_*:* (**a**) parallel direction; (**b**) perpendicular direction; (**c**) module of directions.

Figure 15a–c present the dependence of the total number of events (NoE_TOT_) on ε for close-to-zero positive and negative values of voltage U_g_. It could be assumed that above ε ~ 5%, two NoE_TOT_ values corresponded to a given deformation state, identifying this state unequivocally. This made it possible to develop a solution to an inverse problem—the evaluation of the plastic deformation degree based on the number of total events NoE_TOT_.

**Figure 16:**
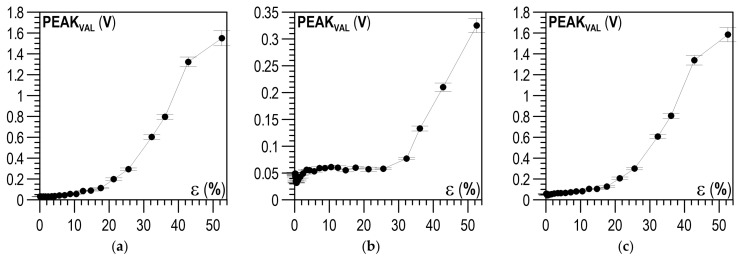
Relation between plastic deformation ε and mean PEAK_VAL_ for descending halves of magnetisation I↘: (**a**) parallel direction; (**b**) perpendicular direction; (**c**) module of directions.

**Figure 17:**
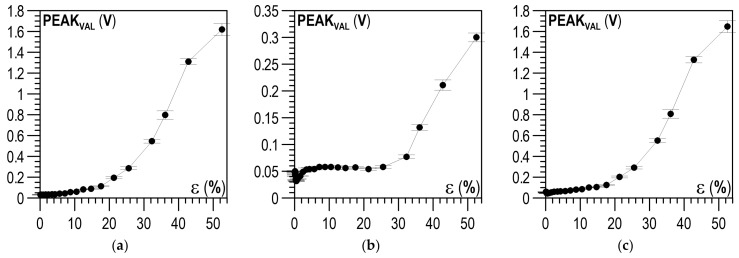
Relation between plastic deformation ε and mean PEAK_VAL_ for ascending halves of magnetisation I↗: (**a**) parallel direction; (**b**) perpendicular direction; (**c**) module of directions.

The analysis then covered the next parameter of the Barkhausen noise: PEAK_VAL_, the values of which were analysed for the descending and ascending halves of the magnetisation. The relations between ε and PEAK_VAL_ for the descending halves of the magnetisation are shown in Figure 16a–c, whereas Figure 17a–c shows the relations for the ascending halves of the magnetisation. For the parallel direction of both halves of the magnetisation (Figure 16a or Figure 17a) and up to about ε = 5%, no clear changes in PEAK_VAL_ values were observed; in the range of ε from 5 to 20%, these changes were slight, after ε = 20% a significant increase in PEAK_VAL_ values was observed. For the perpendicular direction, clear changes could be seen when the plastic deformation degree exceeded ε ~30% (Figure 16b or Figure 17b). Like in the case of U_RMS_, the PEAK_VAL_ module (Figure 16c or Figure 17c) qualitatively and quantitatively resembled the relation for the parallel direction (Figure 16a or Figure 17a).

### 4.4. Metallographic Testing

**Figure 18:**
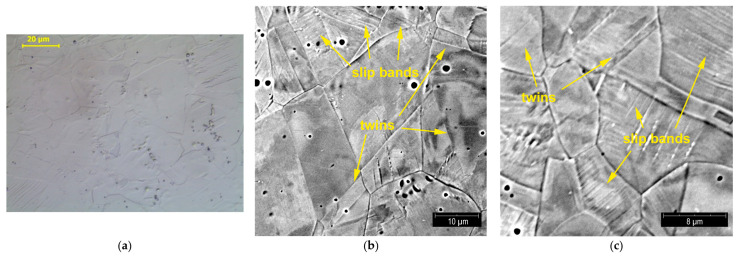
Structure of X2CrNi18-9 steel after 10% plastic deformation; LM (**a**) and SEM (**b**,**c**) observations.

**Figure 19:**
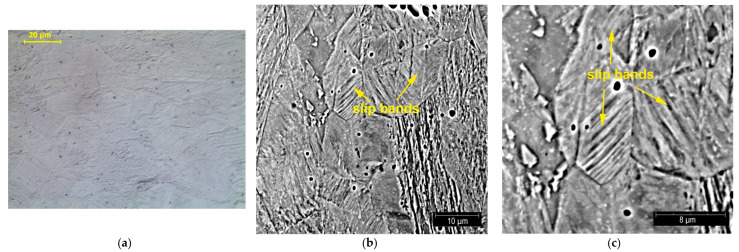
Structure of X2CrNi18-9 steel after 20% plastic deformation; LM (**a**) and SEM (**b**,**c**) observations.

**Figure 20:**
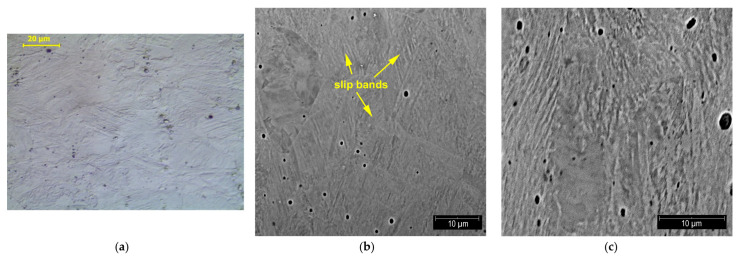
Structure of X2CrNi18-9 steel after 40% plastic deformation; LM (**a**) and SEM (**b**,**c**) observations.

Metallographic tests were carried out to reveal structural changes occurring during the plastic deformation of steel. The three figures below show the structures observed at different degrees of deformation of 10%, 20%, and 40% (Figure 18, Figure 19 and Figure 20, respectively). The observations carried out using light and scanning electron microscopy revealed that the number of structural defects rose with an increase in the deformation degree. The initial structure was austenitic with a small number of twin boundaries (annealing twins)—Figure 5.

A small deformation caused the appearance of slip bands, the density of which increased with a rise in the deformation degree. The formation of the observed slip bands is related to a system within one grain, and the observed effect is the formation of parallel slip faults. The performed observations revealed that, even at 10% deformation of steel, slip bands were arranged in different directions, which indicated that deformation took place in different slip systems—Figure 18.

The highest density of slip bands was obtained at the deformation degree of 40%—Figure 20. A higher degree of deformation was also accompanied by deformation of the grain: i.e., its elongation in the direction of the tensile force.

**Figure 21:**
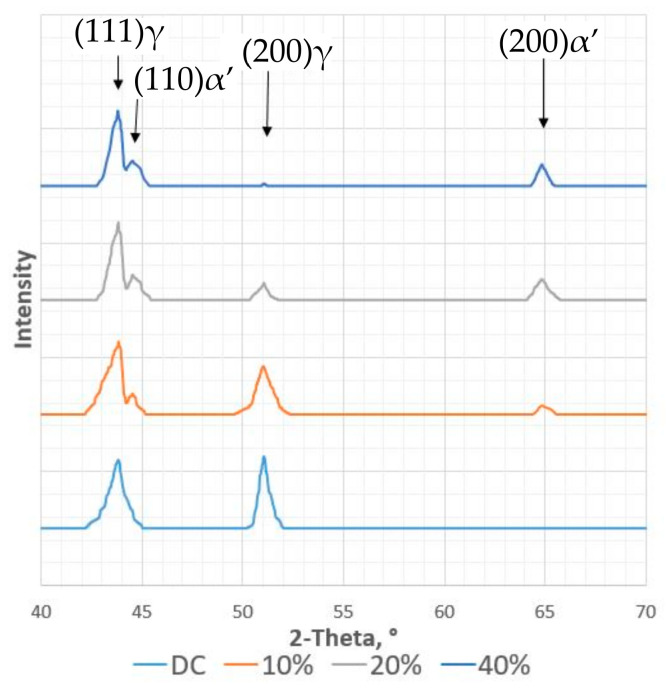
X-ray diffraction patterns of X2CrNi18-9 steel in the initial state (DC) and after selected degrees of deformation (10%, 20%, 40%).

The higher number of defects in the structure caused an increase in the steel hardness, from 280 HV10 for the material in the as-received state to almost 400 HV10 at the 40% deformation degree. On the other hand, no significant increase was observed in the magnetic phase determined using a magnetic field detector, where a value of 0.2 ± 0.1% was recorded for the state at delivery conditions (measurements on specimens after mechanical treatment); after 10% deformation, the magnetic phase increased only up to 1.4 ± 0.1%. An increase in deformation to 20% caused a rise in the magnetic phase content to about 3.0 ± 0.1%, and after 40% deformation to about 14.8 ± 0.1%. The observed increase should be considered to be a value included within the measurement error. The presence of α’, as the effect of deformation, is visible in the XRD analysis. The diffraction lines for the material in the delivery condition (DC) revealed only the γ-phase. Changes could be observed in the diffraction diagrams for the steel post-deformation state in comparison to the DC. An increase in the specimen deformation decreased the intensity of austenite lines, and new martensite lines appeared, whose intensity increased with deformation (Figure 21).

## 5. Summary and Conclusions

The considered issue of the evaluation of plastic deformation based on changes in electromagnetic quantities is one of the so-called inverse problems of non-destructive testing.

Three case studies were presented, in which the following diagnostic signals were used: the residual magnetic field components, the impedance components of the in-series LCR circuit, and the Barkhausen noise features. The changes in microstructure and the resulting changes in electromagnetic properties generated diagnostic signals with an averaged and repeatable value that could be captured using relatively wide-range measuring transducers [42].

Two ranges of strain variability could be distinguished for the extracted features of the diagnostic signals. The ranges differed significantly in the changes in the signal value due to an increment in deformation. Up to the critical plastic deformation degree, the features of diagnostic signals did not show significant changes in value. They could then be related to the content of ferrite δ (chemical composition of steel). However, in the case of deformation higher than critical, where the martensite transformation began, it was possible to use them to evaluate the state of the material. The critical deformation degree determined, based on the performed analyses of diagnostic signals, was as follows: for the RMF ε ~ 10%, the LCR ε ~ 15%, and for the U_RMS_, PEAK_VAL_, and NoE_TOT_ parameters it was ε ~ 20%, ε ~ 20%, and ε ~ 5%, respectively. The differences in the critical deformation value were due to the specificity of the signal individual features. At the same time, they indicated that the changes in the material physical properties (electromagnetic properties, e.g., NoE_TOT_ ε ~ 5%) began much earlier compared to what was suggested by the results of magnetic phase measurements (cf. Table 2). Based on the obtained results, it should be concluded that, depending on the degree of material deformation, different signals should be used for the assessment of the material state.

The NDT results were verified by metallographic examination. In the tested X2CrNi18-9 steel, the ferromagnetic phase—ferrite δ—was present in small amounts already in the non-deformed state. As the deformation increased, and then after a certain critical strain was exceeded, a clear increment in the total share of ferromagnetic phases occurred due to the occurrence of martensite transformation and the formation of martensite α’, as revealed by the XRD and hardness testing.

While typical testing methods showed that the content of the magnetic phase was relatively small even at high deformation (40%), the assessment based on the discussed diagnostic signals made it possible to reveal those changes even for the deformation degree as low as 10–15%. Structural changes (LM and SEM) related to the presence of slip bands were observed already at the deformation degree of 10%, with their density causing significant scattering of diagnostic signals. It was only when the strain exceeded the critical deformation degree of 10% that the martensite transformation (γ → α’) occurred and, thereby, a significant amount of the magnetic phase appeared. In 10% deformation, the content of the magnetic phase increased from 0.2% to 1.4%. This value rose up to 14.8% for 40% deformation. The wide range of results provided by the magnetic field detector led to the conclusion that the content of the magnetic phase strongly depends on the strain rate and the material condition (i.e., chemical composition, heat treatment, residual stress), which indicates that varying conditions of the operating environment can result in different content of the magnetic phase and cause differences in the received diagnostic signals. The presence of the phase, related to the occurrence of the phase transformation, involved the occurrence of signals strong enough to be interpreted and used in diagnostic works aiming to evaluate the state of steel.

The changes in the ferromagnetic phase during plastic deformation of austenitic steels created an opportunity to assess the state of austenitic steel using electromagnetic non-destructive testing methods. The obtained results were unique to the analysed cases. Depending on the properties of the measuring apparatus and its calibration, the initial state of the material, and many other influencing factors [16], the obtained values may differ from those revealed by the tests. The testing results indicated that a further increase in the level of measurement sensitivity in relation to individual diagnostic signals for deformation above the critical deformation degree is of no significance. For deformation lower than critical, further testing and analyses are necessary.

## Figures and Tables

**Table 1:** Chemical composition of X2CrNi18-9 steel (% by mass).

C	Si	Mn	P	S	N	Cr	Mo	Nb	Ni	Ti
0.02	0.45	1.43	0.031	0.008	-	18	0.3	0.017	7.94	0.01

**Table 2:** Magnetic phase content.

Strain ε (%)	No. of Samples	Magnetic Phase Content—Min (%)	Magnetic Phase Content—Max (%)
as-received state	90	0.1	0.2
0–8	31	0.1	0.3
8–15	16	1.1	1.8
15–25	12	2.2	3.9
25–35	14	6.0	9.8
35–45	13	11.6	23.9
45–55	4	25.9	29.3

## Data Availability

The data are available upon request.
